# Knowledge, attitudes and practices about dengue vaccines in Latin America and the Caribbean: a scoping review

**DOI:** 10.1590/0102-311XEN259025

**Published:** 2026-10-12

**Authors:** Miriam Rodfeli Arredondo-Nontol, Denis Garcia-Villegas, Roly Gonzaga-Vilchez, Joan Izquierdo-Marchan, Raquel Aurora Ramos-Salina, Cristhian Junio Castromonte-Izquierdo, Rodolfo Arredondo-Nontol, Narcisa Reto

**Affiliations:** 1 Universidad Nacional de Tumbes, Tumbes, Perú.

**Keywords:** Dengue, Vaccines, Health Knowledge, Attitudes, Practice, Dengue, Vacunas, Conocimientos, Actitudes y Práctica en Salud, Dengue, Vacinas, Conhecimentos, Atitudes e Prática em Saúde

## Abstract

Dengue remains a major public health concern in Latin America and the Caribbean, where vaccination has emerged as a key strategy to reduce severe disease. However, its successful implementation depends on population knowledge, attitudes, and practices (KAP). This study aimed to synthesize available evidence on KAP related to dengue vaccination in the region. A scoping review was conducted following JBI guidelines. Searches were performed in international and regional databases, including MEDLINE/PubMed, Scopus, Embase, Web of Science, SciELO, and the Virtual Health Library. Original studies were included without language or date restrictions. Study selection and data extraction were conducted independently and in duplicate. Sixteen studies were included. Most studies reported limited knowledge regarding vaccine availability, safety, and mechanisms of action. In contrast, attitudes were generally favorable, with high acceptance and willingness to pay, estimated at approximately USD 23 for a three-dose regimen. However, willingness to participate in clinical trials remained low due to safety and trust concerns. Evidence from a study conducted in Puerto Rico indicated that the COVID-19 pandemic was associated with increased intention to receive the dengue vaccine. Practices were less studied, and findings showed low completion of vaccination schedules, indicating a gap between attitudes and uptake. Despite generally favorable attitudes, important knowledge gaps and implementation challenges persist. Strengthening educational strategies and promoting qualified communication between healthcare professionals and the population - particularly during the introduction of new vaccines and in the context of adverse events - may support informed decision-making and improve dengue vaccine uptake.

## Abstract (es)

El dengue sigue siendo una gran preocupación para la salud pública en América Latina y el Caribe, donde la vacunación ha surgido como una estrategia fundamental para reducir las formas graves de la enfermedad. Sin embargo, su implementación exitosa depende del conocimiento, las actitudes y las prácticas (CAP) de la población. Este estudio tuvo como objetivo sintetizar la evidencia disponible sobre CAP de la población relativa a la vacunación contra el dengue en la región. Se realizó una revisión de alcance siguiendo las directrices del JBI. Se realizaron búsquedas en bases de datos internacionales y regionales, entre ellas MEDLINE/PubMed, Scopus, Embase, Web of Science, SciELO y la Biblioteca Virtual en Salud. Se incluyeron estudios sin restricciones de idioma ni fecha de publicación. La selección de los estudios y la extracción de los datos se realizaron de forma independiente y por duplicado. En total, se incluyeron 16 estudios. La mayoría de los estudios reportó un conocimiento limitado sobre la disponibilidad de vacunas, la seguridad y los mecanismos de acción. En cambio, las actitudes fueron generalmente favorables, con una alta aceptación y disposición a pagar, que se estimó en aproximadamente USD 23 para un esquema de tres dosis. Sin embargo, la disposición a participar en ensayos clínicos se mantuvo baja debido a las preocupaciones relacionadas con la seguridad y la confianza. La evidencia de un estudio realizado en Puerto Rico mostró que la pandemia de COVID-19 estuvo asociada con un aumento en la intención de recibir la vacuna contra el dengue. Las prácticas recibieron menor atención en los estudios. Sus resultados mostraron una baja tasa de cumplimiento de los calendarios de vacunación, lo que evidencia una brecha entre las actitudes y la adopción. A pesar de las actitudes generalmente favorables, aún persisten importantes brechas de conocimiento y desafíos en la implementación. Fortalecer las estrategias educativas y fomentar una comunicación de calidad entre los profesionales de la salud y la población -sobre todo durante la introducción de nuevas vacunas y ante eventos adversos- puede favorecer la toma de decisiones informadas y mejorar la adopción de la vacuna contra el dengue.

## Abstract (pt)

A dengue continua sendo uma grande preocupação de saúde pública na América Latina e no Caribe, onde a vacinação surgiu como uma estratégia fundamental para reduzir doenças graves. No entanto, sua implementação bem-sucedida depende de conhecimento, atitudes e práticas (CAP) populacionais. Este estudo buscou sintetizar as evidências disponíveis sobre CAP populacionais relacionadas à vacinação contra a dengue na região. Foi realizada uma revisão de escopo seguindo as diretrizes do JBI. Buscas foram feitas em bancos de dados internacionais e regionais, incluindo MEDLINE/PubMed, Scopus, Embase, Web of Science, SciELO e a Biblioteca Virtual de Saúde. Os estudos foram incluídos sem restrições de idioma ou data. A seleção dos estudos e a extração dos dados foram conduzidas de forma independente e duplicada. No todo, 16 estudos foram incluídos. A maioria dos estudos relatou conhecimento limitado sobre disponibilidade de vacinas, segurança e mecanismos de ação. Em contraste, as atitudes eram geralmente favoráveis, com alta aceitação e disposição para pagar, estimada em aproximadamente USD 23 para um regime de três doses. No entanto, a disposição para participar de ensaios clínicos permaneceu baixa devido a preocupações com segurança e confiança. Evidências de um estudo em Porto Rico indicaram que a pandemia de COVID-19 foi associada a um aumento da intenção de receber a vacina contra a dengue. Práticas foram menos estudadas. Seus resultados mostraram baixa conclusão de calendários de vacinação, indicando uma diferença entre atitudes e adoção. Apesar de atitudes geralmente favoráveis, lacunas importantes de conhecimento e desafios de implementação persistem. Fortalecer estratégias educacionais e promover uma comunicação qualificada entre profissionais de saúde e a população - especialmente durante a introdução de novas vacinas e em eventos adversos - pode apoiar tomadas de decisões informadas e melhorar a adoção da vacina contra a dengue.

## Introduction

Dengue is a viral disease and a major public health concern in more than 125 countries, causing approximately 390 million infections and 500,000 hospitalizations annually, with around 20,000 deaths ^1^. Although most cases are mild, some patients develop severe complications such as capillary leak syndrome and dengue shock, which can be life-threatening ^2^.

Climate change has contributed significantly to the global spread of dengue by expanding the geographic range of *Aedes aegypti* through rising temperatures and changing precipitation patterns ^3^ ^,^ ^4^ ^,^ ^5^. In the absence of specific antiviral treatments, prevention - particularly vaccination - has become a key strategy to reduce severe cases and mortality.

The development of dengue vaccines has taken more than three decades. The tetravalent vaccine CYD-TDV (Dengvaxia; Sanofi, http://www.sanofi.com/), licensed in over 20 countries for individuals aged 9 to 45 years, has shown important limitations. In seronegative individuals, it may increase the risk of severe dengue following natural infection, which has led to restrictions on its use and the need for prior serological screening ^6^ ^,^ ^7^ ^,^ ^8^ ^,^ ^9^.

More recently, a second live-attenuated tetravalent vaccine, TAK-003 (QDenga; Takeda, https://www.takeda.com/), has been approved by international regulatory agencies and introduced in some national immunization strategies. Unlike Dengvaxia, QDenga does not require routine pre-vaccination serological confirmation in its indicated use, although ongoing pharmacovigilance remains essential ^10^.

Post-licensure surveillance systems may detect events supposedly attributable to vaccination or immunization (ESAVI), including rare severe events such as anaphylaxis ^11^. Although uncommon, these events - and how they are communicated - can influence public confidence and acceptance of dengue vaccination ^12^. Given its different implementation requirements, particularly the absence of pre-vaccination screening, QDenga may represent a more scalable option for dengue control programs.

The tetravalent CYD-TDV vaccine (Dengvaxia), based on a yellow fever 17D backbone, has shown limited efficacy against dengue virus serotype 2 (DENV-2) - the most prevalent serotype - and has been associated with an increased risk of severe dengue in seronegative individuals following natural infection. Consequently, its use is currently recommended only in individuals with prior dengue infection confirmed through serological screening ^13^.

In Latin America, where dengue is endemic, vaccination represents a key strategy to reduce severe cases and mortality, and some countries have already incorporated it into national immunization programs. Early indicators such as coverage and schedule completion may also reflect gaps in knowledge, attitudes, and practices (KAP) ^6^ ^,^ ^14^.

Significant gaps persist in public knowledge regarding dengue vaccine safety and efficacy, with up to 50% of the population lacking adequate awareness of its benefits or importance ^15^ ^,^ ^16^ ^,^ ^17^. This situation is further compounded by myths and misinformation, which contribute to vaccine hesitancy ^16^ ^,^ ^17^. Additionally, hesitancy is driven not only by knowledge deficits but also by concerns about ESAVI, levels of trust in health institutions, and shortcomings in risk communication during health crises ^18^.

This scoping review aimed to synthesize the available evidence on KAP regarding dengue vaccination in Latin America and the Caribbean, providing an updated overview and identifying key gaps for public health decision-making.

The following research questions guided this scoping review:

(i) General question: What KAP regarding dengue vaccination have been studied among populations in Latin America and the Caribbean?

(ii) Specific questions: Where do the scientific publications come from and what is their main objective? What types of scientific articles have been published on dengue vaccination, and what do they report about KAP among populations in Latin America and the Caribbean? Which organizations and professional fields are the main authors affiliated with, and who should be contacted for further information about these studies?

## Methods

This scoping review followed the methodological framework established by the JBI ^19^ and the rationale of Arksey & O’Malley ^20^. The review process included five key steps: identifying the research question; identifying relevant studies; selecting studies; charting data; and collating, summarizing, and reporting results. Prior to initiating the review, a protocol was drafted and published in the Open Science Framework ^21^. The *Preferred Reporting Items for Systematic Reviews and Meta-Analyses for Scoping Reviews* (PRISMA-ScR) checklist was used to guide the reporting of this manuscript ^22^.

### Eligibility criteria

Eligibility criteria were based on the Population-Concept-Context (PCC) framework recommended by the JBI.

The population included all individuals residing in Latin America and the Caribbean, with no restrictions on sex, age, education level, or occupation.

The central concept was the evaluation of KAP regarding dengue vaccination.

In this review, “knowledge” referred to factual understanding of dengue vaccination, including availability, effectiveness, safety, indications, and mechanisms of action. “Attitudes” encompassed perceptions, beliefs, concerns, and intentions toward vaccination, whether favorable or unfavorable. “Practices” included observable or self-reported behaviors, such as vaccine uptake, refusal, and adherence to recommendations. These definitions were applied consistently throughout the manuscript to ensure conceptual coherence.

The context focused on original scientific studies - such as clinical trials and observational research - conducted within the Latin American and Caribbean region.

To ensure explicit alignment with the PCC framework, the search strategy incorporated descriptors corresponding to each component: Population (P): populations from Latin America and the Caribbean; Concept (C): KAP regarding dengue vaccination; and Context (C): community and healthcare service settings located in countries of Latin America and the Caribbean.

No limitations were applied regarding language or date of publication.

### Bibliographic search

An exhaustive search was conducted across nine databases: PubMed, Scopus, SciELO, Embase, Google Scholar, Web of Science, the Virtual Health Library (VHL), LILACS, and Redalyc. Controlled vocabulary terms (e.g., *Medical Subject Headings* - MeSH) were combined with free-text keywords and adapted to each database; full strategies are detailed in Supplementary Material (https://cadernos.ensp.fiocruz.br/ojs/index.php/csp/article/view/12107/21105).

In Redalyc, three searches were performed using “dengue” AND “vacuna”, “vacina”, and “vaccine”, screening the first 100 results from each query based on relevance (up to 300 records before duplicate removal). Additionally, reference lists of included studies and relevant reviews were manually screened. The last search date was March 15, 2026.

The search strategy was developed by the research team under the supervision of a senior author with formal training and experience in systematic reviews. Although no information specialist participated, the strategy was internally reviewed and refined to ensure methodological rigor and reproducibility.

### Selection of studies

After retrieval, records were grouped, classified, and imported into Rayyan (https://www.rayyan.ai/), where duplicates were manually removed. Two independent reviewers (D.G.V. and N.R.) screened titles and abstracts in a blinded manner based on predefined inclusion and exclusion criteria.

Full-text articles were subsequently assessed independently and in duplicate by paired reviewers from the research team (R.G.V., J.I.M., R.A.R.S., R.A.N., and M.R.A.N.), following the same criteria. After unblinding, discrepancies were resolved through discussion and consensus, with involvement of a senior author when necessary. Reasons for exclusion at the full-text stage were documented. The study selection process is illustrated in Figure 1.

**Figure 1:**
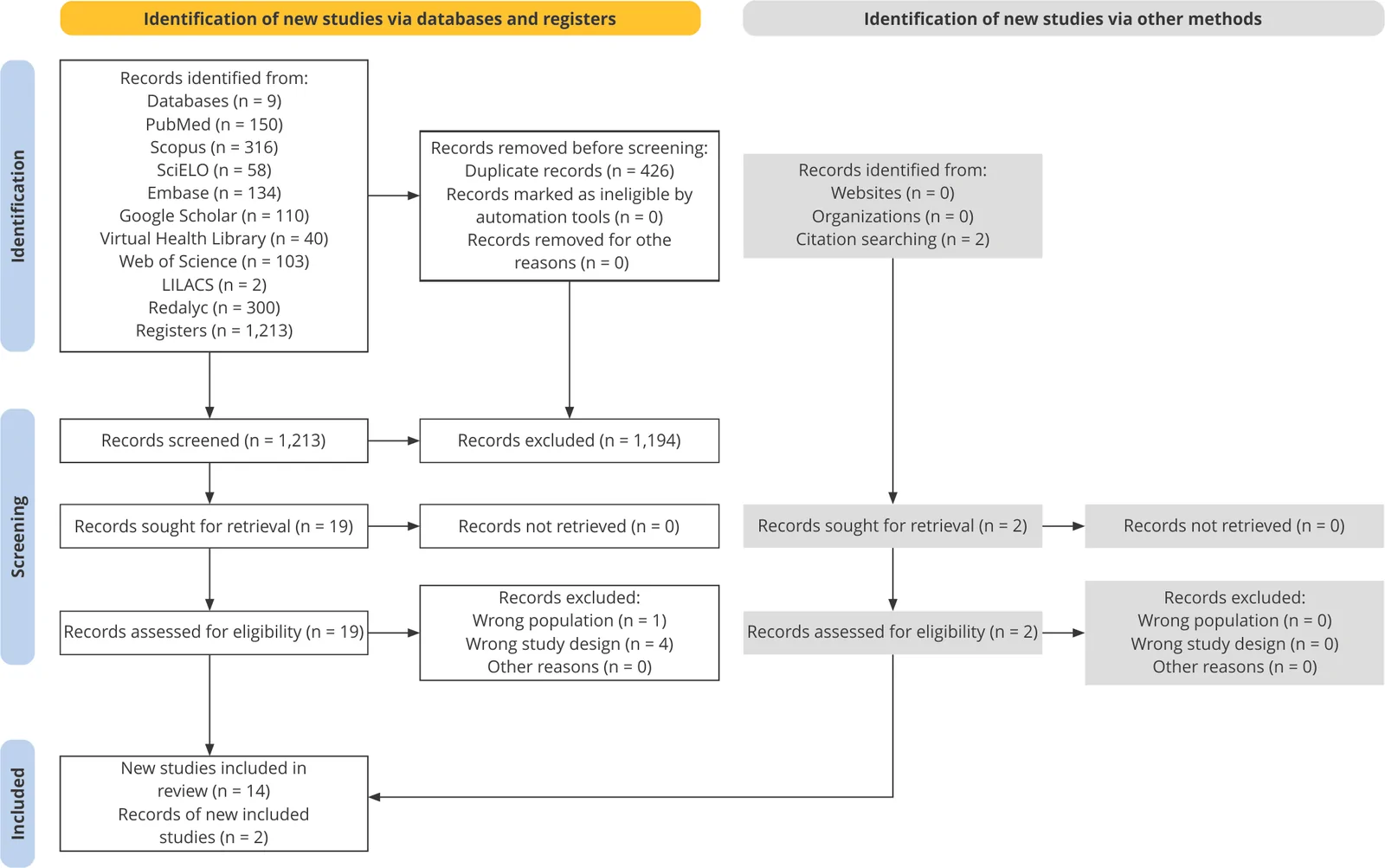
Flow diagram summarizing the study search and selection process.

### Data extraction

After organizing the selected studies in an Excel spreadsheet (https://products.office.com/), a pilot test was conducted with five articles to ensure consistency and accuracy in data extraction, with no discrepancies identified. The extracted data included author, study type, publication year, study characteristics (e.g., location and setting), and population characteristics (e.g., age, gender, and KAP-related variables regarding dengue vaccination).

Data extraction was performed independently and in duplicate by paired reviewers (R.G.V., J.I.M., R.A.R.S., R.A.N., and M.R.A.N.). After unblinding, extracted data were compared, and disagreements were resolved by consensus, with consultation of a senior author (N.R. or D.G.V.) when necessary. No attempts were made to contact study authors for additional information, given the exploratory nature of this scoping review.

### Synthesis of results

The collected data were summarized in tables describing the characteristics of the included studies, complemented by a narrative synthesis of the main findings related to the research question. A scoping review approach was adopted to map the available evidence, categorize studies, and analyze the conceptual focus of the literature, in line with Arksey & O’Malley ^20^. Consistently with this methodology, no formal assessment of risk of bias or study quality was conducted, as the primary objective was to provide an overview of the evidence and identify existing research gaps.

## Results

The systematic search identified a total of 1,213 articles, of which 16 met the inclusion criteria and were included in this scoping review ^14^ ^,^ ^17^ ^,^ ^23^ ^,^ ^24^ ^,^ ^25^ ^,^ ^26^ ^,^ ^27^ ^,^ ^28^ ^,^ ^29^ ^,^ ^30^ ^,^ ^31^ ^,^ ^32^ ^,^ ^33^ ^,^ ^34^ ^,^ ^35^ ^,^ ^36^. Box 1 and the evidence map (Figure 2) summarize the KAP assessed. An expanded version of the details presented in Box 1 is presented in Supplementary Material (https://cadernos.ensp.fiocruz.br/ojs/index.php/csp/article/view/12107/21105).

**Box 1:** Main findings of the articles included in this review.

DIMENSION	STUDIES	MAIN TRENDS	REPRESENTATIVE EXAMPLES
Knowledge	7	A low level of knowledge predominates regarding the vaccine, its efficacy, safety, availability, and eligibility requirements (e.g., seropositivity). Healthcare professionals showed greater familiarity with Dengvaxia, but uncertainties persisted regarding its use and contraindications	^17^ ^,^ ^23^ ^,^ ^25^ ^,^ ^29^ ^,^ ^30^ ^,^ ^31^ ^,^ ^32^
Attitudes	12	Overall, positive attitudes and intention to vaccinate were observed, influenced by risk perception, trust in science/regulators, and COVID-19 experiences. Concerns about adverse events and reluctance to participate in clinical trials persisted; education level and cost modulated acceptance	^17^ ^,^ ^23^ ^,^ ^24^ ^,^ ^26^ ^,^ ^27^ ^,^ ^28^ ^,^ ^30^ ^,^ ^31^ ^,^ ^32^ ^,^ ^34^ ^,^ ^35^ ^,^ ^36^
Practices	4	Vector control measures were maintained despite vaccine availability; real-world programs showed incomplete adherence to the vaccination schedule	^14^ ^,^ ^23^ ^,^ ^32^ ^,^ ^33^

**Figure 2:**
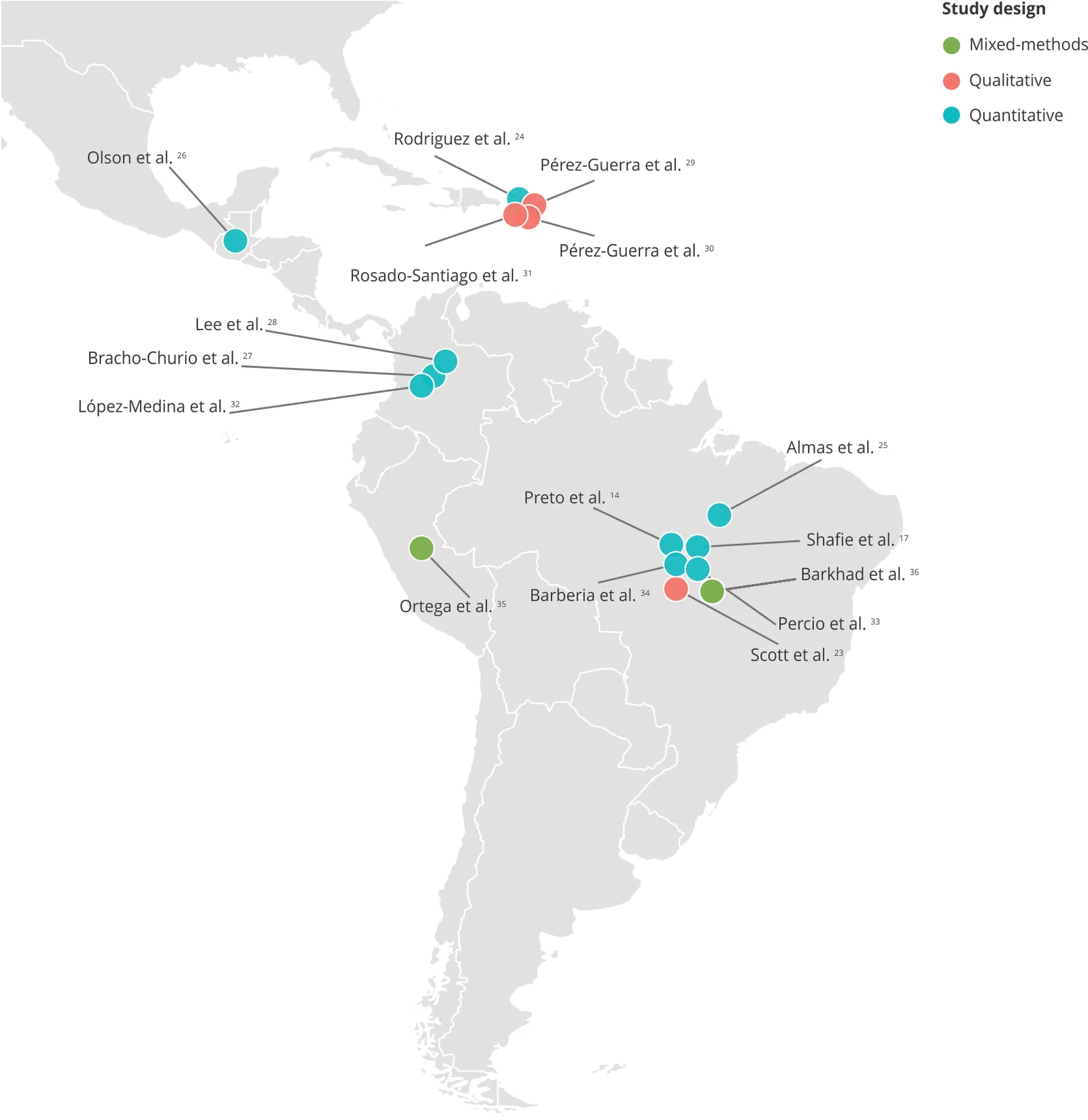
Geographic representation of the included studies and their authors (n = 16). Latin America and the Caribbean.

The included studies were published between 2009 and 2026, reflecting increasing research interest over time, with the highest concentration of publications occurring between 2022 and 2026.

Of the 16 included studies, 4 (25%) used a qualitative design ^23^ ^,^ ^29^ ^,^ ^30^ ^,^ ^31^, 10 (62.5%) employed a quantitative design ^14^ ^,^ ^17^ ^,^ ^24^ ^,^ ^25^ ^,^ ^26^ ^,^ ^27^ ^,^ ^28^ ^,^ ^32^ ^,^ ^33^ ^,^ ^34^, and 2 (12.5%) used mixed-methods with a cross-sectional design ^35^ ^,^ ^36^. Among the quantitative studies, 6 (60%) were cross-sectional studies ^14^ ^,^ ^17^ ^,^ ^25^ ^,^ ^26^ ^,^ ^32^ ^,^ ^34^, 2 (20%) were cohort studies ^24^ ^,^ ^27^, 1 (10%) used predictive analytics ^28^, and 1 (10%) was an ecological cross-sectional study ^33^ (Box 2).

**Box 2:** Characteristics of the included studies (n = 16).

STUDY (YEAR)	POPULATION	DESIGN/METHOD	KAP ASSESSED
López-Medina et al. ^32^ (2026)	Healthcare professionals, 18-65 years, n = 815 (Colombia)	Quantitative - cross-sectional/interview survey	K ✔ A ✔ P ✔
Percio et al. ^33^ (2026)	General population, adolescents 10-14 years, n = 1,907 (Brazil)	Quantitative - ecological cross-sectional	K ✗ A ✗ P ✔
Barberia et al. ^34^ (2025)	Caregivers of children and adolescents ≤ 17 years, n = 779 (Brazil)	Quantitative - cross-sectional/interview survey	K ✗ A ✔ P ✗
Barkhad et al. ^36^ (2025)	General population, adults 18-85 years, n = 167 (Brazil)	Mixed-methods - cross-sectional/interview survey	K ✗ A ✔ P ✗
Ortega et al. ^35^ (2025)	General population, adults > 18 years, n = 147 (Peru)	Mixed-methods - cross-sectional/interview survey	K ✗ A ✔ P ✗
Rosado-Santiago et al. ^31^ (2024)	Parents and healthcare/education professionals, 36-45 years, n = 15 (Puerto Rico)	Qualitative - grounded theory/focus groups + interviews	K ✔ A ✔ P ✗
Scott et al. ^23^ (2023)	General population, 20-70 years, n = 43 (Brazil)	Qualitative - grounded theory/interviews	K ✔A ✔ P ✔
Shafie et al. ^17^ (2023)	General population, 18-60 years, n = 2,400 (Brazil)	Quantitative - cross-sectional/online survey	K ✔ A ✔ P ✗
Rodriguez et al. ^24^ (2023)	Adults > 21 years, n = 2,244 (Puerto Rico)	Quantitative - prospective cohort/interview survey	K ✗ A ✔ P ✗
Almas et al. ^25^ (2022)	Prescribers, 30-60 years, n = 150 (Brazil)	Quantitative - cross-sectional/questionnaire	K ✔ A ✗ P ✗
Preto et al. ^14^ (2020)	General population, 9-44 years, n = 5,000 (Brazil)	Quantitative - descriptive cross-sectional	K ✗ A ✗ P ✔
Olson et al. ^26^ (2018)	Households, n = 564 (Guatemala)	Quantitative - cross-sectional/interviews	K ✗ A ✔ P ✗
Bracho-Churio et al. ^27^ (2017)	General, Indigenous, and Afro-descendant population, 32-54 years, n = 1,037 (Colombia)	Quantitative - population cohort/interviews	K ✗ A ✔ P ✗
Lee et al. ^28^ (2015)	Heads of household, 37-47 years, n = 400 (Colombia)	Quantitative - predictive analytics/interviews	K ✗ A ✔ P ✗
Pérez-Guerra et al. ^29^ (2012)	General population, 9-51 years, n = 326 (Puerto Rico)	Qualitative - grounded theory/focus groups	K ✔ A ✔ P ✗
Pérez-Guerra et al. ^30^ (2009)	Adults > 18 years, n = 59 (Puerto Rico)	Qualitative - grounded theory/focus groups	K ✔ A ✗ P ✗

Note: ✔ = assessed, ✗ = not assessed.

In terms of geographic distribution, the studies originated from Brazil (n = 7), Puerto Rico (n = 4), Colombia (n = 3), Guatemala (n = 1) and Peru (n = 1) (Figure 2).

Four studies were conducted in collaboration with Asian countries also affected by dengue ^17^ ^,^ ^25^ ^,^ ^28^ ^,^ ^32^.

Regarding the study populations, 10 studies focused on the general population ^14^ ^,^ ^17^ ^,^ ^23^ ^,^ ^24^ ^,^ ^28^ ^,^ ^29^ ^,^ ^30^ ^,^ ^33^ ^,^ ^35^ ^,^ ^36^, 3 on prescribers ^25^ ^,^ ^31^ ^,^ ^32^, 1 on a mixed group including general population, Indigenous, and Afro-descendant participants ^27^, 1 on households ^26^, and 1 on adolescents ^34^.

The primary authors of these studies were affiliated with a variety of institutions. Seven studies involved collaborations between universities and pharmaceutical industry clinical research centers ^14^ ^,^ ^17^ ^,^ ^26^ ^,^ ^29^ ^,^ ^30^ ^,^ ^32^ ^,^ ^35^. Three studies were conducted by national disease surveillance centers ^24^ ^,^ ^27^ ^,^ ^33^, while 2 others resulted from inter-university academic collaborations ^19^ ^,^ ^23^. One study was conducted by a pharmaceutical company in the field of pharmacovigilance ^25^, and 3 by independent investigators ^31^ ^,^ ^34^ ^,^ ^36^.

Regarding funding, 7 studies were sponsored by pharmaceutical companies ^17^ ^,^ ^25^ ^,^ ^26^ ^,^ ^27^ ^,^ ^29^ ^,^ ^32^ ^,^ ^35^. Five studies were supported by nonprofit or academic organizations ^23^ ^,^ ^27^ ^,^ ^28^ ^,^ ^34^ ^,^ ^36^. One study was funded by a governmental public health agency ^24^, 1 received independent funding ^31^, and 2 studies did not specify their source of funding ^30^ ^,^ ^33^.

The total number of participants across all studies was 9,017,088, with one additional study that included a survey of 564 households.

The included studies primarily aimed to assess public attitudes toward dengue vaccination - particularly acceptance or rejection - and to identify influencing factors. This encompassed constructs such as willingness to participate in clinical trials, willingness to pay, and knowledge about dengue and its prevention. Some studies also examined the impact of vaccination on vector control practices, while one evaluated the effectiveness of educational interventions for healthcare professionals to support safe vaccine implementation.

### Knowledge

Several studies highlighted limited public knowledge regarding the dengue vaccine and its availability ^17^ ^,^ ^23^ ^,^ ^25^ ^,^ ^29^ ^,^ ^30^ ^,^ ^31^ ^,^ ^32^. A qualitative study reported that, despite general awareness of dengue transmission and prevention, knowledge about dengue vaccines - particularly regarding safety, efficacy, and regulatory approval - was limited ^23^. Similarly, a cross-sectional study in South America and Asia found that vaccine-related knowledge remained below 30% in countries such as Argentina, Brazil, Colombia, and Mexico ^17^.

In Puerto Rico, although some participants recognized potential benefits of vaccination, understanding of vaccine characteristics, risks, and eligibility criteria was still limited ^29^. These findings suggest insufficient access to clear and reliable information. Additionally, qualitative studies indicated low awareness of vaccine availability, with some participants perceiving dengue as more severe due to the absence of a known vaccine ^30^.

Regarding prescriber knowledge, one study on Dengvaxia was very limited: only 3.3% demonstrated correct understanding. Although most knew it is indicated only for previously infected individuals, major gaps remained - few recognized the risk in seronegative patients, appropriate diagnostic methods, or correct eligibility criteria, with frequent errors across specialties ^25^.

Similarly, one study reported a moderate level of knowledge among healthcare professionals, with a composite score of 56/100, despite high self-confidence in their understanding of vaccine safety, efficacy, dosing, and patient counseling ^32^.

In another qualitative study, key informants were generally familiar with Dengvaxia and its dosing regimen; however, important uncertainties persisted regarding interactions with comorbidities, co-administration with other vaccines, timing after dengue infection, and administration details. Awareness remained uneven: while pediatricians and researchers were familiar with related clinical trials, only a few parents had heard of the vaccine, and familiarity among parents and school personnel was limited ^31^.

### Attitudes

Twelve studies explored public attitudes toward dengue vaccination ^17^ ^,^ ^23^ ^,^ ^24^ ^,^ ^26^ ^,^ ^27^ ^,^ ^28^ ^,^ ^30^ ^,^ ^31^ ^,^ ^32^ ^,^ ^34^ ^,^ ^35^ ^,^ ^36^.

Most participants reported positive attitudes toward receiving the dengue vaccine, although their acceptance was often conditional on the perception that the vaccine was both safe and effective.

While specific definitions of safety and efficacy were not always provided by participants, many emphasized the need for a high level of confidence in these attributes before accepting the vaccine ^23^.

Only one study reported overall low attitudes toward dengue vaccination, with no country exceeding 66% acceptance; higher educational levels were associated with more favorable attitudes ^36^. Although attitudes toward vaccination were generally higher than knowledge levels, they remained lower than attitudes toward dengue disease and its prevention (60% vs. 70% and 76%, respectively) ^17^.

Vaccine acceptance was strongly influenced by perceived efficacy: 75% of participants were willing to accept a vaccine with 50% efficacy, increasing to 88% at 75% efficacy ^26^. Acceptance was higher among individuals in densely populated areas, those receiving information from healthcare professionals, and certain parental characteristics, while reliance on public healthcare services was associated with lower acceptance. Additionally, one study found that vaccine demand was inversely related to educational level ^28^.

A qualitative study showed divergent opinions regarding willingness to receive the dengue vaccine, with responses primarily influenced by participants’ level of vaccine knowledge. Some individuals expressed a willingness to be vaccinated if their serological test results confirmed previous dengue infection. Others rejected the vaccine due to doubts about its safety and efficacy, while a third group remained undecided but expressed interest in obtaining more information before making a decision ^31^. Consistently with these trends, a mixed-methods study in Peru reported a high acceptability of dengue vaccination (81.2%) ^35^. However, vaccine hesitancy was significantly associated with negative perceptions of COVID-19 immunization (OR = 2.52), indicating a persistent impact of the pandemic on institutional trust. Additional factors associated with greater hesitancy included limited knowledge of dengue transmission (OR = 3.34), higher or technical education (OR = 6.48), preference for natural immunity (OR = 2.46), higher socioeconomic status, and urban residence. In contrast, willingness to pay for the vaccine (OR = 0.37) and preference for door-to-door vaccination strategies (OR = 0.54) were associated with lower hesitancy ^35^.

Key informants, including healthcare and education professionals, showed generally positive attitudes toward recommending Dengvaxia, supported by greater familiarity with the vaccine and active participation in vaccination campaigns ^31^. Similarly, another study reported a favorable attitude score among healthcare professionals (70/100), with perceived urgency to protect patients and confidence in vaccines associated with higher likelihood of recommendation ^32^.

In school-based settings, 13.6% of caregivers would not allow dengue vaccination, a proportion comparable to HPV and influenza vaccines but lower than COVID-19, suggesting similar acceptance to other routine vaccines ^34^.

A mixed-methods study also reported moderately positive attitudes, with about two-thirds recognizing the vaccine’s protective effect. Favorable attitudes were associated with ages 26-35 years and higher education level, while self-employment was associated with lower acceptance; qualitative findings indicated general willingness despite concerns about limited availability ^36^.

A study in dengue-endemic Peruvian cities found 81.2% willingness to vaccinate, higher in Iquitos and among males. Acceptance was driven by information on safety, efficacy, dosing, and manufacturer, as well as recommendations from healthcare professionals and approval by the Peruvian Ministry of Health. Lower acceptance in Piura was associated with distrust in authorities and perceptions of government neglect ^35^.

### Intention to vaccinate and the impact of COVID-19

The COVID-19 pandemic was associated with increased acceptance of dengue vaccination, with adult intention rising from 73.4% to 84.5% and parental intention from 75.6% to 85.5% ^24^. Concerns about side effects and distrust decreased, although the demand for more information and consultation with healthcare professionals increased, particularly among parents. Overall, intention to vaccinate was significantly higher after the pandemic (adjusted OR: 2.27 in adults and 2.21 in parents). Higher acceptance was associated with prior influenza vaccination, frequent mosquito exposure, and male sex, while being self-employed or a student was associated with lower intention; no significant differences were observed by age, race, education, or income.

### Attitudes toward clinical trials

A 2012 study evaluating public attitudes toward dengue vaccine clinical trials found generally positive perceptions of vaccines, particularly regarding their role in preventing severe dengue. However, willingness to participate in trials or enroll children was low, mainly due to distrust in new vaccines and trial procedures, fear of side effects or infection, and insufficient information about vaccine candidates ^29^.

### Willingness to pay

One study reported an inverse relationship between vaccine cost and willingness to pay, with higher prices associated with lower acceptance ^27^. Greater willingness to pay was observed among individuals perceiving a higher risk of dengue or with a prior history of infection.

Another study estimated an average willingness to pay at approximately USD 23 for a three-dose vaccine, with most participants preferring lower price ranges and only a minority willing to pay higher amounts ^28^. Similarly, 41.2% of participants in another study were willing to pay, although most considered vaccination should be publicly funded; acceptable price ranges ranged between PEN 5 and 50 (USD 1.40-13.60) ^35^.

Higher willingness to pay was associated with maternal education and prior vaccine purchase, while lower willingness was associated with larger household size and higher dengue-related knowledge ^28^.

### Practices

Four studies examined practices related to dengue vaccination ^14^ ^,^ ^23^ ^,^ ^32^ ^,^ ^33^. Vector control behaviors were generally maintained after vaccination, although adherence appeared influenced by perceived risk and confidence in vaccine effectiveness.

A large-scale study by Preto et al. ^14^ (n = 302,603) showed high initial uptake but low completion of the three-dose schedule, with 52.8% failing to complete it; 60% received at least one dose and 44.2% at least two doses. Uptake was higher in earlier campaign phases, among males, and in younger age groups (9-14 and 28-44 years), while adherence was lowest in the 25-27 age group ^14^.

Among healthcare professionals, a high engagement in discussing and recommending vaccination was observed (score 72/100); however, a gap persisted between recommendation and uptake, as vaccination was discussed in 44.8% of consultations but administered in only 27.2% ^32^.

## Discussion

Dengue has a long history in Latin America and the Caribbean, with recurrent outbreaks documented across the region for centuries ^37^. Given the sustained burden of dengue in the region, several studies have explored population knowledge regarding the disease, its transmission, clinical manifestations, and preventive measures ^17^.

Significant knowledge gaps persist regarding dengue prevention through vaccination ^17^. This scoping review found that the general population in Latin America and the Caribbean has limited understanding of vaccine availability, mechanisms of action, and safety ^17^ ^,^ ^23^ ^,^ ^25^ ^,^ ^29^ ^,^ ^30^ and that even approved vaccines such as Dengvaxia remain largely unknown in the region ^38^.

These findings are consistent with studies conducted in other endemic regions such as Asia ^39^ ^,^ ^40^. However, rather than reflecting a general lack of dengue awareness, the observed knowledge gap appears to represent a structural separation between historically dominant vector-control paradigms and the more recent introduction of vaccination as a complementary preventive strategy ^17^. In contrast, studies conducted in parts of Asia where higher levels of knowledge were reported linked this outcome to better educational attainment, higher income levels, and prior personal or family experience with dengue infection ^41^, highlighting the role of broader social determinants.

Two studies conducted before the U.S. Food and Drug Administration (FDA) approval of Dengvaxia highlighted limited public information about the vaccine, which likely contributed to later attitudinal concerns ^29^ ^,^ ^30^. Dengue was widely perceived as a serious disease, but vaccination acceptance depended on better information about risks and adverse effects. More recent evidence shows persistent uncertainty, especially among parents, whose willingness to vaccinate their children is often conditional on clearer information regarding approval processes, safety, adverse events, pre-vaccination testing, and insurance coverage ^31^.

Although Dengvaxia was initially well received, its use became controversial due to limited efficacy in seronegative individuals and the risk of severe dengue ^7^. Errors in pre-vaccination screening can lead to iatrogenic harm, underscoring the need for strict adherence to eligibility criteria and effective risk communication to maintain public trust ^42^.

Therefore, healthcare professionals must have strong knowledge of vaccine indications to avoid prescription errors, while future vaccination strategies should address both knowledge gaps and attitudinal factors, including risk perception and trust, as well as counter misinformation ^43^.

Although most studies were conducted during the Dengvaxia context, the dengue vaccination landscape is rapidly evolving, with newer vaccines such as QDenga (TAK-003) already approved and implemented in some countries, including Brazil ^44^, and others such as the Instituto Butantan vaccine showing promising phase-3 results ^45^.

Findings should be interpreted within a changing vaccination context, as KAP may vary according to each vaccine’s characteristics. No study evaluated KAP regarding TAK-003, likely due to its recent introduction, underscoring the need for further research ^46^.

Additionally, among healthcare providers, knowledge of dengue vaccines and their indications remains limited, with notable prescription errors - especially in travelers and adults in endemic areas without confirmed infection ^25^.

One study reported adequate knowledge among healthcare professionals regarding clinical trials, dosing, and complications of Dengvaxia ^31^; however, gaps between knowledge and practice persist. Despite frequent vaccine discussions (44.8%), administration rates remain lower (27.2%), indicating barriers in translating recommendations into uptake ^33^.

Furthermore, knowledge alone is insufficient for vaccine acceptance, as cognitive and emotional factors - such as risk perception, fear of adverse events, trust in healthcare systems, and prior experiences - also influence decisions ^18^ ^,^ ^47^. Health literacy plays a key role in linking knowledge to action, facilitating informed decision-making, while limited literacy increases vulnerability to misinformation and reduces adherence ^48^ ^,^ ^49^.

Knowledge gaps among healthcare professionals may hinder dengue vaccination program implementation, highlighting the need for continuing education and targeted training on vaccine indications, eligibility, and risk management to prevent errors and strengthen risk communication and public trust ^50^ ^,^ ^51^.

Assessing and improving vaccine-related knowledge is essential, as it is closely associated with population acceptance, requiring efforts at both community and healthcare provider levels to ensure successful immunization strategies ^38^ ^,^ ^52^.

Vaccine acceptance can be explained by frameworks such as the Health Belief Model and the Theory of Planned Behavior, which emphasize factors such as perceived risk, benefits, barriers, and social norms ^53^. However, in Latin America, decisions are also informed by social context, collective experiences, and trust in health institutions, where risk communication and institutional trust play a key role ^54^ ^,^ ^55^.

These perspectives help explain variability across studies and support the development of context-sensitive vaccination policies and communication strategies. Most studies reported generally favorable attitudes toward dengue vaccination ^23^ ^,^ ^24^ ^,^ ^28^ ^,^ ^30^, although one study found low levels of positive attitude ^17^.

In contrast, studies from Indonesia reported poor attitudes in up to 70% of participants, associated with low education, agricultural occupation, low socioeconomic status, and limited KAP ^56^. However, other evidence showed that higher education and rural residence were also associated with higher vaccine hesitancy, highlighting variability in determinants ^35^.

While regional differences must be acknowledged, these findings demonstrate the need to tailor vaccination programs to local contexts, as public acceptance is crucial for successful implementation ^57^. Positive attitudes toward vaccines are strongly associated with higher uptake ^58^.

This review found that acceptance was related to perceived efficacy and safety, with an inverse relationship with educational level ^23^ ^,^ ^26^ ^,^ ^28^. Similarly, studies from Indonesia identified gender, income, socioeconomic status, and positive attitudes toward dengue prevention as factors associated with higher vaccine acceptance ^59^.

The COVID-19 pandemic was associated with increased acceptance of dengue vaccination and reduced concerns about side effects and mistrust ^23^. However, this effect appears context-dependent, as some populations showed greater confidence while others became more polarized, and in certain settings vaccination decisions remained unchanged ^24^ ^,^ ^60^.

Despite generally favorable attitudes, willingness to participate in clinical trials remains limited, mainly due to fear of adverse effects and distrust in research processes ^29^. Additional barriers include fear of infection, lack of time, and distrust of pharmaceutical companies, which represents a key obstacle to vaccination uptake and large-scale immunization efforts ^61^.

These barriers limit participation in clinical research and hinder the generation of evidence on vaccine safety and efficacy. Strategies such as crowdsourcing have been proposed to improve public engagement and willingness to participate ^62^ ^,^ ^63^. Addressing hesitancy also requires public education, transparent communication of risks and benefits, and strengthened trust in health institutions ^23^.

Additionally, cost represents a key barrier to vaccine uptake, as willingness to pay decreases with increasing price ^26^ ^,^ ^27^ ^,^ ^28^. Similar findings have been reported in Asia ^64^ ^,^ ^65^, and, in low-income countries, demand is high mainly when vaccines are affordable or government-subsidized ^41^.

Willingness to pay for dengue vaccination is influenced by prior dengue experience, socioeconomic and educational level, perceived risk and severity, and previous vaccine behavior ^26^ ^,^ ^27^ ^,^ ^28^. Similar determinants have been reported in Asia, including knowledge, positive attitudes, preventive practices, employment status, and prior exposure ^43^ ^,^ ^66^, with higher income associated with greater willingness to pay ^40^.

However, the acceptable payment threshold in Latin America and the Caribbean (up to USD 23) is lower than that reported in meta-analyses and Asian studies ^67^ ^,^ ^68^, likely reflecting differences in income, healthcare system access, and outbreak experience ^69^. This underscores the need for financing and subsidy strategies to ensure equitable vaccine access.

Only two studies addressed vaccination practices, showing that, although initial acceptance was high, completion of the full three-dose schedule was lower than expected ^23^ ^,^ ^26^. This dropout highlights an important operational challenge and raises concerns about the sustainability of multi-dose vaccination strategies, particularly in settings with limited follow-up, suggesting that simplified dosing schedules could improve adherence. Similar issues have been reported in other multi-dose programs ^70^.

Adherence varied by demographic factors, with higher rates in men and in individuals aged 9-14 years and 28-44 years, and the highest dropout among those aged 25-27 years ^14^. Evidence from Asia also associates adherence with knowledge, education, and prior dengue infection ^39^.

Despite vaccination, most individuals would continue vector control practices ^14^; however, there is concern that vaccines with limited efficacy could create a false sense of protection and reduce these measures, potentially increasing transmission ^71^.

This review has several limitations, including the absence of methodological quality assessment, risk-of-bias analysis, and quantitative synthesis, which limits the strength of conclusions. The small number and heterogeneity of studies, along with their concentration in Brazil and Puerto Rico (69%), also restrict representativeness and generalizability. Additionally, limited evidence on vaccination practices constrained deeper analysis of this dimension.

Despite this, the findings have key implications for immunization strategies. Addressing knowledge gaps among communities and healthcare providers requires structured educational interventions and transparent risk communication on vaccine efficacy, safety, and eligibility ^72^ ^,^ ^73^. Ensuring equitable access demands public financing and inclusion in national insurance systems ^74^. Strengthening the role of healthcare professionals as trusted intermediaries is essential to improve decision-making and reduce misinformation ^75^, while context-sensitive communication strategies that consider local risk perceptions and institutional trust are crucial to sustain vaccine uptake ^31^.

## Conclusion

This scoping review synthesized evidence on KAP regarding dengue vaccination in Latin America and the Caribbean. Sixteen studies were included ^14^ ^,^ ^17^ ^,^ ^23^ ^,^ ^24^ ^,^ ^25^ ^,^ ^26^ ^,^ ^27^ ^,^ ^28^ ^,^ ^29^ ^,^ ^30^ ^,^ ^31^ ^,^ ^32^ ^,^ ^33^ ^,^ ^34^ ^,^ ^35^ ^,^ ^36^.

Overall, knowledge about dengue vaccines was limited, particularly regarding availability, safety, and mechanisms of action ^17^ ^,^ ^23^ ^,^ ^25^ ^,^ ^29^ ^,^ ^30^. In contrast, attitudes were generally favorable, with high acceptance and a willingness to pay estimated at approximately USD 23 for a three-dose regimen ^26^ ^,^ ^28^, although willingness to participate in clinical trials remained low due to concerns about safety and trust ^29^.

Evidence also suggests increased vaccine acceptance following the COVID-19 pandemic ^23^ ^,^ ^24^. However, practices were less frequently studied, and available findings indicate suboptimal completion of vaccination schedules, highlighting a gap between positive attitudes and actual uptake ^14^ ^,^ ^26^ ^,^ ^33^.

These findings highlight the need for educational strategies to improve knowledge, as well as strengthened and qualified communication between healthcare professionals and the population, particularly during the introduction of new vaccines and in the context of adverse events following immunization.

## Data Availability

The sources of information used in the study are indicated in the body of the article.
