# Supplementary material for: Knowledge, attitudes and practices about dengue vaccines in Latin America and the Caribbean: a scoping review
Source: Cad Saude Publica. 2026 Oct 12;42:e00259025. doi: 10.1590/0102-311XEN259025 (PMC13661011; doi:10.1590/0102-311XEN259025)
Supplement: Supplementary file 1 [file 1678-4464-csp-42-EN259025-s.pdf]

SUPPLEMENTARY MATERIAL

Supplementary Material 1 Search strategies employed for the databases searched for this scoping review.

Date of search: March 15, 2026

Filters: None

Databases	Set	Search terms	Items found
PUBMED	1	“Dengue Vaccines” [Mesh] OR “Dengue Vaccine*” [tiab] OR “Dengue Tetravalent Vaccine, Live” [tiab] OR “Tetravalent Dengue Vaccine” [tiab] OR “Dengvaxia Vaccine” [tiab] OR “qdenga” [tiab]	150
	2	Knowledge[Mesh] OR Knowledge[tiab]	
	3	“Health Knowledge, Attitudes, Practice” [Mesh] OR “Health Knowledge, Attitudes, Practice” [tiab]	
	4	Attitudes [Mesh] OR Opinion* [tiab] OR Sentiment* [tiab]	
	5	Practice [tiab] OR Practice [tw]	
	6	Perception [Mesh] OR Sensory Processing[tiab] OR Perception*[tiab]	
	7	("Latin America"[MeSH Terms] OR "Latin America"[Title/Abstract] OR "Latin America"[Other Term] OR "Hispano America"[Title/Abstract] OR "latin*"[Title/Abstract] OR "latin*"[Other Term] OR "hispan*"[Title/Abstract] OR "hispan*"[Other Term] OR "Central America"[MeSH Terms] OR "Central America"[Title/Abstract] OR "Central America"[Other Term] OR "Caribbean Region"[MeSH Terms] OR "caribbean*"[Title/Abstract] OR "caribbean*"[Other Term] OR "South America"[MeSH Terms] OR "South America"[Title/Abstract] OR "South America"[Other Term] OR "mexico"[MeSH Terms] OR "mexic*"[Title/Abstract] OR "mexic*"[Other Term] OR "Belize"[MeSH Terms] OR "Belize"[Title/Abstract] OR "Belize"[Other Term] OR "Belice"[Title/Abstract] OR "Belice"[Other Term] OR "British Honduras"[Title/Abstract] OR "Costa Rica"[MeSH Terms] OR "costa rica*"[Title/Abstract] OR "costa rica*"[Other Term] OR "El Salvador"[MeSH Terms] OR "El Salvador"[Title/Abstract] OR "El Salvador"[Other Term] OR "guatemala"[MeSH Terms] OR "guatemal*"[Title/Abstract] OR "guatemal*"[Other Term] OR "honduras"[MeSH Terms] OR "hondura*"[Title/Abstract] OR "hondura*"[Other Term] OR "nicaragua"[MeSH Terms] OR "nicaragua*"[Title/Abstract] OR "nicaragua*"[Other Term] OR "panama"[MeSH Terms] OR "panama*"[Title/Abstract] OR "panama*"[Other Term] OR "West Indies"[MeSH Terms] OR "West Indies"[Title/Abstract] OR "West Indies"[Other Term] OR "Caribbean Islands"[Title/Abstract] OR "Caribbean Islands"[Other Term] OR "Montserrat"[Title/Abstract] OR "Montserrat"[Other Term] OR "Turks and Caicos Islands"[Title/Abstract] OR "Turks and Caicos Islands"[Other Term] OR "Cayman Islands"[Title/Abstract] OR "Cayman Islands"[Other Term] OR "Antilles"[Title/Abstract] OR "Antilles"[Other Term] OR "Leeward Islands"[Title/Abstract] OR "Leeward Islands"[Other Term] OR "Windward Islands"[Title/Abstract] OR "Windward Islands"[Other Term] OR "Caribbean Region"[MeSH Terms] OR "Caribbean"[Title/Abstract] OR "Caribbean"[Other Term] OR "Caribbean Netherlands"[MeSH Terms] OR "anguilla*"[Title/Abstract] OR "anguilla*"[Other Term] OR "anguila*"[Title/Abstract] OR "anguila*"[Title/Abstract] OR "Antigua and Barbuda"[MeSH Terms] OR "Barbuda"[Title/Abstract] OR "Barbuda"[Other Term] OR "aruba"[MeSH Terms] OR "aruba*"[Title/Abstract] OR "aruba*"[Other Term] OR "bahamas"[MeSH Terms] OR "baham*"[Title/Abstract] OR "baham*"[Other Term] OR "Barbados"[MeSH Terms] OR "Barbados"[Title/Abstract] OR "Barbados"[Other Term] OR "Bonaire"[Title/Abstract] OR "Bonaire"[Other Term] OR "Sint Eustatius"[Title/Abstract] OR "Saba"[Title/Abstract] OR "Saba"[Other Term] OR "cuba"[MeSH Terms] OR "cuba*"[Title/Abstract] OR "cuba*"[Other Term] OR "Curacao"[MeSH Terms] OR "Curacao"[Title/Abstract] OR "Curacao"[Other Term] OR "Curazao"[Title/Abstract] OR "dominica"[MeSH Terms] OR "dominica*"[Title/Abstract] OR "dominica*"[Other Term] OR "Grenada"[Title/Abstract] OR "Grenada"[Other Term] OR "granada*"[Title/Abstract] OR "granada*"[Other Term] OR "guadeloupe"[MeSH Terms] OR "guadeloupe*"[Title/Abstract] OR "guadeloupe*"[Other Term] OR "guadalupe*"[Title/Abstract] OR "guadalupe*"[Other Term] OR "haiti"[MeSH Terms] OR "haiti*"[Title/Abstract] OR "haiti*"[Other Term] OR "Virgin Islands"[Title/Abstract] OR "Virgin Islands"[Other Term] OR "Virgenes"[Title/Abstract] OR "British Virgin Islands"[MeSH Terms] OR "United States Virgin	

		Islands"[MeSH Terms] OR "jamaica"[MeSH Terms] OR "jamaica*"[Title/Abstract] OR "jamaica*"[Other Term] OR "Martinique"[MeSH Terms] OR "Martinique"[Title/Abstract] OR "Martinique"[Other Term] OR "Martinica"[Title/Abstract] OR "Martinica"[Other Term] OR "Puerto Rico"[MeSH Terms] OR "puerto ric*"[Title/Abstract] OR "puerto ric*"[Other Term] OR "Dominican Republic"[MeSH Terms] OR "Dominican Republic"[Title/Abstract] OR "Dominican Republic"[Other Term] OR "Republica Dominicana"[Title/Abstract] OR "Republica Dominicana"[Other Term] OR "San Bartolome"[Title/Abstract] OR "Saint Kitts and Nevis"[MeSH Terms] OR "st kitts and nevis"[Title/Abstract] OR "Kitts"[Title/Abstract] OR "Kitts"[Other Term] OR "Nevis"[Title/Abstract] OR "Nevis"[Other Term] OR "saint vincent and the grenadines"[MeSH Terms] OR "st vincent and the grenadines"[Title/Abstract] OR "st vincent and the grenadines"[Other Term] OR "Grenadines"[Title/Abstract] OR "Grenadines"[Other Term] OR "Saint Lucia"[MeSH Terms] OR "Saint Lucia"[Title/Abstract] OR "Saint Lucia"[Other Term] OR "st lucia"[Title/Abstract] OR "st lucia"[Other Term] OR "Santa Lucia"[Title/Abstract] OR "Santa Lucia"[Other Term] OR "Sint Maarten"[MeSH Terms] OR "Sint Maarten"[Title/Abstract] OR "Sint Maarten"[Other Term] OR "Saint Martin"[Title/Abstract] OR "Saint Martin"[Other Term] OR "San Martin"[Title/Abstract] OR "San Martin"[Other Term] OR "Trinidad and Tobago"[MeSH Terms] OR "Trinidad"[Title/Abstract] OR "Trinidad"[Other Term] OR "Tobago"[Title/Abstract] OR "Tobago"[Other Term] OR "argentina"[MeSH Terms] OR "argentin*"[Title/Abstract] OR "argentin*"[Other Term] OR "bolivia"[MeSH Terms] OR "bolivia*"[Title/Abstract] OR "bolivia*"[Other Term] OR "brazil"[MeSH Terms] OR "brazil*"[Title/Abstract] OR "brazil*"[Other Term] OR "brasil*"[Title/Abstract] OR "brasil*"[Other Term] OR "chile"[MeSH Terms] OR "chile*"[Title/Abstract] OR "chile*"[Other Term] OR "colombia"[MeSH Terms] OR "colombia*"[Title/Abstract] OR "colombia*"[Other Term] OR "ecuador"[MeSH Terms] OR "ecuador*"[Title/Abstract] OR "ecuador*"[Other Term] OR "Malvinas"[Title/Abstract] OR "Malvinas"[Other Term] OR "guyana*"[MeSH Terms] OR "guyana*"[Title/Abstract] OR "Guyana"[Other Term] OR "Guiana"[Title/Abstract] OR "Guiana"[Other Term] OR "French Guiana"[MeSH Terms] OR "paraguay"[MeSH Terms] OR "paraguay*"[Title/Abstract] OR "paraguay*"[Other Term] OR "peru"[MeSH Terms] OR "peru*"[Title/Abstract] OR "peru*"[Other Term] OR "suriname"[MeSH Terms] OR "surinam*"[Title/Abstract] OR "surinam*"[Other Term] OR "uruguay"[MeSH Terms] OR "uruguay*"[Title/Abstract] OR "uruguay*"[Other Term] OR "venezuela"[MeSH Terms] OR "venezuela*"[Title/Abstract] OR "venezuela*"[Other Term])	
SCOPUS	1	TITLE-ABS-KEY ("dengue vaccines" OR "dengue vaccine*" OR "dengue tetravalent vaccine, live" OR "tetravalent dengue vaccine" OR "dengvaxia vaccine" OR qdenga)	316
	2	TITLE-ABS-KEY (knowledge OR knowledge)	
	3	TITLE-ABS-KEY ("health knowledge, attitudes, practice" OR "health knowledge, attitudes, practice")	
	4	TITLE-ABS-KEY (attitudes OR opinion* OR sentiment*)	
	5	TITLE-ABS-KEY (practice OR practice)	
	6	(TITLE-ABS-KEY (perception OR perceptio*)	
	7	TITLE-ABS-KEY (("latin america" OR "latin america" OR "latin america[other term]" OR "hispano america" OR latin* OR "latin*[other term]" OR hispan* OR "hispan*[other term]" OR "central america" OR "central america" OR "central america[other term]" OR "caribbean region" OR caribbean* OR "caribbean*[other term]" OR "south america" OR "south america" OR "south america[other term]" OR mexico OR mexic* OR "mexic*[other term]" OR belize OR belize OR "belize[other term]" OR belice OR "belice[other term]" OR "british honduras" OR "costa rica" OR "costa rica*" OR "costa rica*[other term]" OR "el salvador" OR "el salvador" OR "el salvador[other term]" OR guatemala OR guatemal* OR "guatemal*[other term]" OR honduras OR hondura* OR "hondura*[other term]" OR nicaragua OR nicaragua* OR "nicaragua*[other term]" OR panama OR panama* OR "panama*[other term]" OR "west indies" OR "west indies" OR "west indies[other term]" OR "caribbean islands" OR "caribbean islands[other term]" OR montserrat OR "montserrat[other term]" OR "turks and caicos islands" OR "turks and caicos islands[other term]" OR "cayman islands" OR "cayman islands[other term]" OR antilles OR "antilles[other term]" OR "leeward islands" OR "leeward islands[other term]" OR "windward islands" OR "windward islands[other term]" OR "caribbean region" OR caribbean OR "caribbean[other term]" OR "caribbean netherlands" OR anguilla* OR "anguilla*[other term]" OR anguila* OR anguila* OR "antigua and barbuda" OR barbuda OR "barbuda[other term]" OR aruba OR aruba* OR "aruba*[other term]" OR bahamas OR baham* OR "baham*[other term]" OR barbados OR barbados OR "barbados[other term]" OR bonaire OR "bonaire[other term]" OR "sint eustatius" OR saba OR "saba[other term]" OR cuba OR cuba* OR "cuba*[other term]" OR curacao OR curacao OR "curacao[other term]" OR curazao OR dominica OR dominica* OR "dominica*[other term]" OR grenada OR "grenada[other term]" OR granada* OR "granada*[other term]" OR guadeloupe OR guadeloupe* OR	

		"guadeloupe*[other term]" OR guadalupe* OR "guadalupe*[other term]" OR haiti OR haiti* OR "haiti*[other term]" OR "virgin islands" OR "virgin islands[other term]" OR virgenes OR "british virgin islands" OR "united states virgin islands" OR jamaica OR jamaica* OR "jamaica*[other term]" OR martinique OR martinique OR "martinique[other term]" OR martinica OR "martinica[other term]" OR "puerto rico" OR "puerto ric*" OR "puerto ric*[other term]" OR "dominican republic" OR "dominican republic" OR "dominican republic[other term]" OR "republica dominicana" OR "republica dominicana[other term]" OR "san bartolome" OR "saint kitts and nevis" OR "st kitts and nevis" OR kitts OR "kitts[other term]" OR nevis OR "nevis[other term]" OR "saint vincent and the grenadines" OR "st vincent and the grenadines" OR "st vincent and the grenadines[other term]" OR grenadines OR "grenadines[other term]" OR "saint lucia" OR "saint lucia" OR "saint lucia[other term]" OR "st lucia" OR "st lucia[other term]" OR "santa lucia" OR "santa lucia[other term]" OR "sint maarten" OR "sint maarten" OR "sint maarten[other term]" OR "saint martin" OR "saint martin[other term]" OR "san martin" OR "san martin[other term]" OR "trinidad and tobago" OR trinidad OR "trinidad[other term]" OR tobago OR "tobago[other term]" OR argentina OR argentin* OR "argentin*[other term]" OR bolivia OR bolivia* OR "bolivia*[other term]" OR brazil OR brazil* OR "brazil*[other term]" OR brasil* OR "brasil*[other term]" OR chile OR chile* OR "chile*[other term]" OR colombia OR colombia* OR "colombia*[other term]" OR ecuador OR ecuador* OR "ecuador*[other term]" OR malvinas OR "malvinas[other term]" OR guyana* OR guyana* OR "guyana[other term]" OR guiana OR "guiana[other term]" OR "french guiana" OR paraguay OR paraguay* OR "paraguay*[other term]" OR peru OR peru* OR "peru*[other term]" OR suriname OR surinam* OR "surinam*[other term]" OR uruguay OR uruguay* OR "uruguay*[other term]" OR venezuela OR venezuela* OR "venezuela*[other term]"))	
SCIELO	1	(Dengue Vaccines)OR(Dengue Vaccine*) OR (Dengue Tetravalent Vaccine, Live) OR (Tetravalent Dengue Vaccine) OR (Dengvaxia Vaccine) OR (qdenga)	58
	2	(knowledge) OR (health knowledge, attitudes, practice) OR (attitudes) OR (opinion*)OR (sentiment*)	
EMBASE	1	('South and Central America'/exp OR 'Latin America' OR 'South and Central America' OR 'america, south and central') AND ('dengue vaccine'/exp OR 'dengue tetravalent vaccine' OR 'dengue vaccine' OR 'dengue vaccines' OR 'dengue virus vaccine' OR 'dengue virus vaccines' OR 'dengvaxia' OR 'denvax' OR 'qdenga' OR 'tak 003' OR 'tak003' OR 'tetravax-dv') AND (attitude/exp OR 'attitude' OR 'behavior, permissive' OR 'behaviour, permissive' OR 'faculty attitude' OR 'knowledge, attitudes, practice' OR 'permissive behavior' OR 'permissive behaviour' OR 'permissiveness' OR 'rejection (psychology)' OR 'rejection, psychology')	134
	2	('dengue vaccine'/exp OR 'dengue tetravalent vaccine' OR 'dengue vaccine' OR 'dengue vaccines' OR 'dengue virus vaccine' OR 'dengue virus vaccines' OR 'dengvaxia' OR 'denvax' OR 'qdenga' OR 'tak 003' OR 'tak003' OR 'tetravax-dv') AND ('perception'/exp OR 'perception' OR 'perception size' OR 'perceptual performance' OR 'perceptual processing' OR 'perceptual task' OR 'size detection' OR 'size discrimination' OR 'size perception' OR 'social perception' OR 'weight perception')	
GOOGLE SCHOLAR	1	(Dengue Vaccines)OR(Dengue Vaccine*) OR (Dengue Tetravalent Vaccine, Live) OR (Tetravalent Dengue Vaccine) OR (Dengvaxia Vaccine) OR (qdenga) AND (knowledge) OR (health knowledge, attitudes, practice) OR (attitudes) OR (opinion*)OR (sentiment*) AND ((Argentina*) OR (Bolivia*) OR (Brazil*) OR (Brasil*) OR (Chile*) OR (Colombia*) OR (Ecuador*) OR (Malvinas) OR (Guyana) OR (“French Guiana”) OR (Paraguay*) OR (Peru*) OR (Surinam*) OR (Uruguay*) OR (Venezuela*))	110
VIRTUAL HEALTH LIBRARY	1	(Dengue Vaccines) OR (Dengue Vaccine*) OR (Dengue Tetravalent Vaccine, Live) OR (Tetravalent Dengue Vaccine) OR (Dengvaxia Vaccine) OR (qdenga)	40
	2	(Knowledge) OR (Knowledge)	
	3	(Health Knowledge, Attitudes, Practice) OR (Health Knowledge, Attitudes, Practice)	
	4	(attitudes) OR (opinion) OR (Sentiment*)	
	5	(Practice) OR (Practice)	
	6	(Perception) OR (Sensory Processing) OR (Perception*)	
	7	(Argentina*) OR (Bolivia*) OR (Brazil*) OR (Brasil*) OR (Chile*) OR (Colombia*) OR (Ecuador*) OR (Malvinas) OR (Guyana) OR (French Guiana) OR (Paraguay*) OR (Peru*) OR (Surinam*) OR (Uruguay*) OR (Venezuela*) OR (Latin America) OR (Latin American countries) OR (developing countries)	

WEB OF SCIENCE	1	TS=(Practice OR Practice OR Attitudes OR Opinion* OR Sentiment* OR "Health Knowledge, Attitudes, Practice")	103
	2	Dengue Vaccines OR Dengue Vaccine* OR Dengue Tetravalent Vaccine, Live OR Tetravalent Dengue Vaccine OR Dengvaxia Vaccine OR qdenga	
	3	"Latin America" OR "Hispano America" OR Latin* OR Hispan* OR "Central America" OR Caribb* OR "South America" OR Mexic* OR Belize OR Belice OR "British Honduras" OR "Costa Rica*" OR "El Salvador" OR Guatemala* OR Hondur* OR Nicaragua* OR Panama OR "West Indies" OR "Caribbean Islands" OR Montserrat OR "Turks and Caicos Islands" OR "Cayman Islands" OR Antilles OR "Leeward Islands" OR "Windward Islands" OR “Caribbean Region” OR Anguilla OR Anguila OR “Antigua and Barbuda” OR Antigua OR Barbuda OR Aruba OR Bahamas OR Barbados OR Bonaire OR "Sint Eustatius" OR Saba OR Cuba* OR Curacao OR Curazao OR Dominica* OR Grenada OR Granada OR Guadeloupe OR Guadalupe OR Haiti* OR "Virgin Islands" OR Virgenes OR Jamaica OR Martinique OR Martinica OR "Puerto Ric*" OR "Dominican Republic" OR "Republica Dominicana" OR "San Bartolome" OR "Saint Kitts and Nevis" OR “St. Kitts and Nevis” OR Kitts OR Nevis OR "Saint Vincent and the Grenadines" OR "St. Vincent and the Grenadines" OR Grenadines OR "Saint Lucia" OR "St. Lucia" OR "Santa Lucia" OR "Sint Maarten" OR "Saint Martin" OR "San Martin" OR “Trinidad and Tobago” OR Trinidad OR Tobago OR Argentin* OR Bolivia* OR Brazil* OR Brasil* OR Chile* OR Colombia* OR Ecuador* OR Malvinas OR Guyana* OR Paraguay* OR Peru* OR Surinam* OR Uruguay* OR Venezuela*) OR TI=("Latin America" OR "Hispano America" OR Latin* OR Hispan* OR "Central America" OR Caribb* OR "South America" OR Mexic* OR Belize OR Belice OR "British Honduras" OR "Costa Rica*" OR "El Salvador" OR Guatemala* OR Hondur* OR Nicaragua* OR Panama OR "West Indies" OR "Caribbean Islands" OR Montserrat OR "Turks and Caicos Islands" OR "Cayman Islands" OR Antilles OR "Leeward Islands" OR "Windward Islands" OR “Caribbean Region” OR Anguilla OR Anguila OR “Antigua and Barbuda” OR Antigua OR Barbuda OR Aruba OR Bahamas OR Barbados OR Bonaire OR "Sint Eustatius" OR Saba OR Cuba* OR Curacao OR Curazao OR Dominica* OR Grenada OR Granada OR Guadeloupe OR Guadalupe OR Haiti* OR "Virgin Islands" OR Virgenes OR Jamaica OR Martinique OR Martinica OR "Puerto Ric*" OR "Dominican Republic" OR "Republica Dominicana" OR "San Bartolome" OR "Saint Kitts and Nevis" OR “St. Kitts and Nevis” OR Kitts OR Nevis OR "Saint Vincent and the Grenadines" OR "St. Vincent and the Grenadines" OR Grenadines OR "Saint Lucia" OR "St. Lucia" OR "Santa Lucia" OR "Sint Maarten" OR "Saint Martin" OR "San Martin" OR “Trinidad and Tobago” OR Trinidad OR Tobago OR Argentin* OR Bolivia* OR Brazil* OR Brasil* OR Chile* OR Colombia* OR Ecuador* OR Malvinas OR Guyana* OR Paraguay* OR Peru* OR Surinam* OR Uruguay* OR Venezuela*) OR AB=("Latin America" OR "Hispano America" OR Latin* OR Hispan* OR "Central America" OR Caribb* OR "South America" OR Mexic* OR Belize OR Belice OR "British Honduras" OR "Costa Rica*" OR "El Salvador" OR Guatemala* OR Hondur* OR Nicaragua* OR Panama OR "West Indies" OR "Caribbean Islands" OR Montserrat OR "Turks and Caicos Islands" OR "Cayman Islands" OR Antilles OR "Leeward Islands" OR "Windward Islands" OR “Caribbean Region” OR Anguilla OR Anguila OR “Antigua and Barbuda” OR Antigua OR Barbuda OR Aruba OR Bahamas OR Barbados OR Bonaire OR "Sint Eustatius" OR Saba OR Cuba* OR Curacao OR Curazao OR Dominica* OR Grenada OR Granada OR Guadeloupe OR Guadalupe OR Haiti* OR "Virgin Islands" OR Virgenes OR Jamaica OR Martinique OR Martinica OR "Puerto Ric*" OR "Dominican Republic" OR "Republica Dominicana" OR "San Bartolome" OR "Saint Kitts and Nevis" OR “St. Kitts and Nevis” OR Kitts OR Nevis OR "Saint Vincent and the Grenadines" OR "St. Vincent and the Grenadines" OR Grenadines OR "Saint Lucia" OR "St. Lucia" OR "Santa Lucia" OR "Sint Maarten" OR "Saint Martin" OR "San Martin" OR “Trinidad and Tobago” OR Trinidad OR Tobago OR Argentin* OR Bolivia* OR Brazil* OR Brasil* OR Chile* OR Colombia* OR Ecuador* OR Malvinas OR Guyana* OR Paraguay* OR Peru* OR Surinam* OR Uruguay* OR Venezuela*) OR AK=("Latin America" OR "Hispano America" OR Latin* OR Hispan* OR "Central America" OR Caribb* OR "South America" OR Mexic* OR Belize OR Belice OR "British Honduras" OR "Costa Rica*" OR "El Salvador" OR Guatemala* OR Hondur* OR Nicaragua* OR Panama OR "West Indies" OR "Caribbean Islands" OR Montserrat OR "Turks and Caicos Islands" OR "Cayman Islands" OR Antilles OR "Leeward Islands" OR "Windward Islands" OR “Caribbean Region” OR Anguilla OR Anguila OR “Antigua and Barbuda” OR Antigua OR Barbuda OR Aruba OR Bahamas OR Barbados OR Bonaire OR "Sint Eustatius" OR Saba OR Cuba* OR Curacao OR Curazao OR Dominica* OR Grenada OR Granada OR Guadeloupe OR Guadalupe OR Haiti* OR "Virgin Islands" OR Virgenes OR Jamaica OR Martinique OR Martinica OR "Puerto Ric*" OR "Dominican Republic" OR "Republica Dominicana" OR "San Bartolome" OR "Saint Kitts and Nevis" OR “St. Kitts and Nevis” OR Kitts OR Nevis OR "Saint Vincent and the Grenadines" OR "St. Vincent and the Grenadines" OR Grenadines OR "Saint Lucia" OR "St. Lucia" OR "Santa Lucia" OR "Sint Maarten" OR "Saint Martin" OR "San Martin" OR “Trinidad and Tobago” OR Trinidad OR Tobago OR Argentin* OR Bolivia* OR Brazil* OR Brasil* OR Chile* OR Colombia* OR Ecuador* OR Malvinas OR Guyana* OR Paraguay* OR Peru* OR Surinam* OR Uruguay* OR Venezuela*)	

LILACS		(dengue) AND (vacuna OR vacina OR vaccine OR immunization OR inmunizacion OR imunização) AND (conocimiento OR conhecimento OR knowledge OR actitudes OR atitudes OR attitudes OR practicas OR práticas OR practices OR percepcion OR percepção OR perception)	2
REDALYC		“dengue” AND “vacuna”, “dengue” AND “vacina” e “dengue” AND “vaccine”	300

Supplementary Material 2 Supplementary Details of the Main Conclusions Presented in Table 1.

	Knowledge		Attitudes		Practices	
	**Content**	**Main findings**	**Content**	**Main findings**	**Content**	**Main findings**
Scott V 2023(17)	Yes	Lack of vaccine knowledge, efficacy and safety	Yes	The high willingness to be vaccinated against dengue was based on perceived susceptibility to the virus, previous negative experiences, desire for protection, and confidence in science and regulatory processes.	Yes	Most participants will continue with vector control practices, considering these measures essential even with vaccination, as they believe that no vaccine offers complete protection, and their practices would be based on previous experiences with the COVID-19 vaccine.
ShafieA 2023(12)	Yes	Participants were unaware of the vaccine and its availability.	Yes	Participants showed a low positive attitude toward vaccines. Educational level was positively associated with more favorable attitudes.	No	
Rodríguez D.M. 2023(18)	No		Yes	After COVID-19, the number of questions about adverse events of the dengue vaccine among adult respondents as well as parents was reduced. However, the number of parents who needed more information about the vaccine increased. Dengue vaccination intention increased in both adult respondents and parents, being higher in parents who received influenza vaccine in the previous year or who reported frequent mosquito bites. On the other hand, parents who were working or studying had a lower intention of vaccinating their children. No significant differences were found by sex, age, race, education or income.	No	
Almas M 2022(19)	Yes	Knowledge of the tetravalent vaccine was low. Only 11.3% identified the need to confirm seropositivity by blood test, and few knew that vaccination is not recommended for persons traveling to endemic areas from non-endemic areas. Although almost all participants correctly identified patients eligible for vaccination, most also considered incorrect scenarios as eligible, such as patients without clinical support or with negative results for previous infection.	No		No	
Preto C. 2021(20)	No		No		Yes	During the vaccination program, 302,603 people received at least one dose. Sixty percent of the population-initiated vaccination, but only 44.2% completed two doses. Males showed higher adherence than females. The groups aged 28 to 44 years and 9 to 14 years had higher uptake, while

						those aged 25 to 27 years had higher dropout (59.7%). Overall, 52.8% did not complete three doses, with the lowest dropout in children aged 9 to 14 years (33.2%).
Olson D 2018(21)	No		Yes	Attitudes toward vaccines were mostly positive. Intention to be vaccinated against DENV increased with perceived efficacy. Factors such as living in dense areas, having older children and receiving medical information favored acceptance, while public health care reduced it. The majority had a willingness to pay less than US$4 for the vaccine, being higher in families with a higher educational level of the mother, but lower in those with more children and better knowledge about DENV.	No	
Bracho Churio YT 2017(22)	No		Yes	Willingness to pay for the vaccine decreased with increasing cost. Single-dose vaccines were preferred over three-dose schedules and those with greater efficacy and duration. In addition, willingness to pay was higher in those who perceived high risk of contracting dengue or had a history of the disease.	No	
Lee J 2015	No		Yes	Demand for the dengue vaccine increases with economic income, although in Colombia interest was lower among the more educated. The perceived seriousness of the disease was a key factor in its acquisition, more than the risk of contagion. In the three countries studied, vaccination of young children was prioritized, with a willingness to pay $23 for three doses, reflecting a high interest in immunization as protection, despite the lack of details on its safety.	No	
Perez Guerra C 2012	Yes	Participants lacked specific knowledge about dengue vaccines and clinical trials. Although they recognized their usefulness in preventing disease, some associated vaccines with the use of the causative virus and with side effects. Some parents considered a dengue vaccine beneficial but expressed the need to know the risks and adverse effects before deciding, reflecting their lack of	Yes	The attitude is mostly positive because of the preventive potential against serious dengue diseases, but participants refuse to participate in clinical trials or to include their children in them.	No	

		prior information.				
Pérez Guerra C 2009	Yes	Dengue is considered a serious disease due to the absence of a vaccine to prevent the disease.	No		No	
Rosado Santiago C 2024	Yes	While most professionals knew about Dengvaxia's dosage and risks, they and parents still questioned its interactions and administrative details. Surprisingly, awareness of the clinical trials remained very low, reaching only a few specialists and a handful of parents.	Yes	Vaccination opinions were split based on personal knowledge: some accepted it only if seropositive, while others remained uncertain or skeptical due to safety concerns. Conversely, health and education professionals were highly supportive, actively recommending the Dengvaxia® vaccine and offering to lead public immunization campaigns.	No	
Lopez-Medina E 2026	Yes	Healthcare professionals showed moderate dengue vaccine knowledge (56/100), scoring lower in this area than in attitudes or practices. Despite this, they reported high self-perceived expertise regarding vaccine safety, efficacy, dosing, and patient education.	Yes	Healthcare professionals generally support dengue vaccination (70/100), driven by patient protection urgency and vaccine confidence. These positive attitudes significantly increase their likelihood of recommending the vaccine to patients.	Yes	Healthcare professionals show high engagement in promoting the dengue vaccine with a 72/100 score; however, a significant gap remains as only 27.2% of discussed cases result in actual administration.
Percio J 2026	No		No		Yes	Dengue vaccination coverage remains low and inconsistent, averaging 29.76% with most municipalities under 50%. This trend is driven by delivery methods, vaccine hesitancy, logistics, and poor record-keeping.
Barberia L 2025	No		Yes	The study found that 13.6% of caregivers would refuse school-based dengue vaccination, a rate comparable to HPV and influenza but significantly lower than the 21.11% refusal rate for COVID-19. This suggests that school-based dengue immunization faces similar acceptance levels as other routine vaccines.	No	
Ortega E 2025	No		Yes	Data shows high acceptance (81.2%) for a dengue vaccine, driven by trust in health authorities and fear of severe disease. However, hesitancy persists among urban, higher-income groups due to technical misinformation and a preference for natural immunity. To ensure success, participants recommend house-to-house distribution to avoid long waits and suggest tiered pricing or gratuity based on the COVID-19 experience.	No	
Barkhad A 2025	No		Yes	While attitudes toward dengue vaccination were generally positive, with	No	

				two-thirds of participants recognizing its protective benefits, support was strongest among individuals aged 26–35 and those with university degrees. Conversely, independent workers showed less favorable views, and despite a broad willingness to vaccinate, older participants expressed frustration regarding limited vaccine accessibility.		

LEGEND	
	General population
	General population, indigenous and Afro-descendant populations
	Heads of household
	Health providers
